# An Optimized Approach to Perform Bone Histomorphometry

**DOI:** 10.3389/fendo.2018.00666

**Published:** 2018-11-21

**Authors:** Deeksha Malhan, Matthias Muelke, Sebastian Rosch, Annemarie B. Schaefer, Felix Merboth, David Weisweiler, Christian Heiss, Ignacio Arganda-Carreras, Thaqif El Khassawna

**Affiliations:** ^1^Experimental Trauma Surgery, Faculty of Medicine, Justus-Liebig University of Giessen, Giessen, Germany; ^2^Department of Trauma, Hand, and Reconstructive Surgery, University Hospital of Giessen and Marburg, Giessen, Germany; ^3^Department of Computer Science and Artificial Intelligence, Basque Country University, San Sebastian, Spain

**Keywords:** bone histomorphometry, open source, imageJ, Trainable Weka Segmentation, BoneJ, bone area, fracture healing

## Abstract

Bone histomorphometry allows quantitative evaluation of bone micro-architecture, bone formation, and bone remodeling by providing an insight to cellular changes. Histomorphometry plays an important role in monitoring changes in bone properties because of systemic skeletal diseases like osteoporosis and osteomalacia. Besides, quantitative evaluation plays an important role in fracture healing studies to explore the effect of biomaterial or drug treatment. However, until today, to our knowledge, bone histomorphometry remain time-consuming and expensive. This incited us to set up an open-source freely available semi-automated solution to measure parameters like trabecular area, osteoid area, trabecular thickness, and osteoclast activity. Here in this study, the authors present the adaptation of Trainable Weka Segmentation plugin of ImageJ to allow fast evaluation of bone parameters (trabecular area, osteoid area) to diagnose bone related diseases. Also, ImageJ toolbox and plugins (BoneJ) were adapted to measure osteoclast activity, trabecular thickness, and trabecular separation. The optimized two different scripts are based on ImageJ, by providing simple user-interface and easy accessibility for biologists and clinicians. The scripts developed for bone histomorphometry can be optimized globally for other histological samples. The showed scripts will benefit the scientific community in histological evaluation.

## Introduction

Disease diagnostics in preclinical and clinical research relies on several methods like histology, radiology, gene expression, and blood serum analysis. The application of such method integrates together to provide a comprehensive set of biological information (1). Histological examination is one of the gold standards for the diagnosis of infectious diseases (2). Whereas, in bone research, radiological testing are used as gold standard to diagnose bone fractures or bone loss. Nonetheless, histology, and histomorphometry serve as powerful tool in assessing systemic skeletal diseases like osteoporosis (3). Histomorphometry is one of the standard method to study different cell type activities under normal and diseased condition. The scientific community provided a standardized nomenclature and method to evaluate bone parameters according to the American Society of Bone and Mineral Research (ASBMR) (1, 4, 5). The application of bone histomorphometry with molecular data analysis benefited in understanding cellular discrepancies in systemic skeletal diseases (6).

The advancement of computational techniques promoted the development of commercial as well as freely available image processing programs and softwares. Previous studies reported the application of different softwares to automate bone histomorphometry (7–11). Such programs were set up on commercial platforms such as Matlab and Visiopharm (7, 9). The high cost of commercially available products limits the application of software in worldwide scientific community.

Evaluation of osteoblast and osteoclast activity was reported in some studies (7, 11) while other reported the evaluation of features like bone area and cartilage area using animal and human samples (8, 9). Nonetheless, van't Hof et al. recently reported the application of java based script compatible with freely available open-source ImageJ software to analyze osteoblast and osteoclast activity (11). They aimed to perform histomorphometric assessment of bone resorption, osteoid, and fluorochrome labeled samples.

ImageJ was developed at the NIH and is a leading platform that provides different software package for image analysis (12). ImageJ provides several different features such as osteoclast length and cell count to examine cellular changes in diseased model. Arganda-Carreras et al. developed a user-friendly Trainable Weka Segmentation (TWS) plug-in compatible with ImageJ to perform quantitative segmentation of microscope images (13). Doube et al. developed an open-source ImageJ based plugin; BoneJ to analyze standard bone measurements from computed tomography scanned images (14).

This study adopted the TWS, ImageJ, and BoneJ libraries to perform bone histomorphometry following ASBMR guidelines from simple histological stain like Von Kossa/Van Gieson to complex histological stain like Movat Pentachrome. Besides, the set up scripts were tested on immunohistochemical stained sections. This study set up a freely available and user-friendly scripts to perform semi-automated bone histomorphometry. The established scripts were used on different histological stains and bone samples.

## Methods

### Materials and equipment

Bone sections (see section “Sample collection and preparation”).

Histology (see section “Histological stain”).

Light microscope (see section “Image capturing”).

32-bit/64-bit based operating system (equipped with Java, see section “Software”).

Fiji ImageJ (see section “Software”).

### Ethical statement

The protocol describes an optimized approach to perform quantitative evaluation of histological sections. The histological sections were obtained from osteoporotic animal model. The animal experiments were performed in full agreement with the Institutional laws and the German animal protection laws. All experiments were approved by the ethical commission of the local governmental institution [“Regierungspraesidium Darmstadt,” permit no. Gen. Nr. F31/36 (sheep)] and [“Regierungspraesidium Giessen,” permit no. Gen. Nr. 20/10-Nr.A31/2009 (rat)].

### Sample collection and preparation

The samples were obtained from ovariectomized female Merino Land Sheep of average age 5.5 years and Sprague-Dawley rats of age 2.5 months. Both the rat and sheep animal models were established to study osteoporosis as described before (15–17). Iliac crest biopsy samples from sheep study and lumbar vertebral (L1) samples from rat study were collected after euthanasia and freed from muscles. Sheep samples were then embedding in Poly-Methyl-Metha-Acrylate (PMMA; Technovit® 9100, Heraeus Kulzer, Hanau, Germany) using standardized protocol (18). Rat samples were fixed in 4% paraformaldehyde (PFA) and later decalcified using 4% PFA and 14% Ethylenediaminetetraacetic acid (EDTA) at 4°C for 4 weeks. Undecalcified sheep embedded samples were cut into 5 μm thick sections onto Kawamoto's film (Section-Lab Co. Ltd., Hiroshima, Japan). Decalcified paraffin embedded rat samples were cut into 6 μm thick slices. The sections were obtained using a motorized rotary microtome (Thermo/Microm HM 355 S, Thermo Scientific GmbH, Karlsruhe, Germany).

### Histological stain

Decalcified and undecalcified histological stains were carried out to explore structural and cellular changes in the different animal models. PMMA embedded sections were used to carry out Von Kossa/Van Gieson stain and Movat Pentachrome. While, paraffin embedded sections were used to carry out immunohistochemical (IHC) stains like Osteocalcin and histochemical stain like Tartrate Resistant Acid Phosphatase (TRAP).

#### Von Kossa/Van Gieson staining

Von Kossa/Van Gieson stain was used to distinguish the mineralized bone matrix from non-mineralized bone matrix. The stain distinguishes mineralized bone matrix in black and non-mineralized bone matrix in red color. The staining protocol was carried out as described before (19).

#### Movat pentachrome staining

Movat Pentachrome stain was used to visualize various constituents of a connective tissue. The stain distinguishes the tissues so mineralized bone appears bright yellow, mineralized cartilage appears blue-green, non-mineralized cartilage appear yellow, non-mineralized bone, elastic fibers, and muscles appear bright red. The staining protocol was adapted from previous study (20).

#### Osteocalcin IHC

Osteocalcin is a known biological marker to explore bone formation. Therefore, osteocalcin IHC was carried to analyze osteoblast activity. The staining protocol was adapted from previous study (21).

#### TRAP enzyme histochemistry

TRAP is a known biological marker to examine bone resorption process. Therefore, TRAP enzyme histochemistry was carried to analyze osteoclast activity. The staining protocol was adapted from a previous study (17).

### Image capturing

Images were taken using a Leica microscopy system (Leica DM5500 photomicroscope equipped with a DFC7000 camera and operated by LASX software version 3.0, Leica Microsystem Ltd, Wetzlar, Germany). Von Kossa/Van Gieson and Movat Pentachrome stained sections were imaged at 5X (0.77 pixel/μm) magnification. Osteocalcin IHC stained sections were imaged at 10X (1.55 pixel/μm) magnification. TRAP stained sections were imaged at 40X (6.17 pixel/μm) magnification.

### Software

The success of the established protocol requires a 32/64-bit operating system. The scripts relies on java and Fiji ImageJ. Therefore, any operating system (Windows/Mac/Linux) with updated version of java can be used to perform histomorphometry. The Fiji ImageJ (version 1.51r; NIH, Maryland, USA) was used as a platform to run the program. The open source software project TWS (13) was used as the base to create an optimized script to get bone parameters like mineralized area, trabecular area. While, BoneJ (14) was used as the base to create an optimized second script to obtain parameters like trabecular thickness and trabecular separation. The optimized TWS script was written in BeanShell while the optimized BoneJ script was written in Java.

### Reproducibility and validation

The inter-observer differences in measurements generated by TWS were assessed. The differences were assessed by comparing the analyses of hematoxylin stained rat samples (*n* = 8) carried out by two users independently. Additionally, GNU Image Manipulation Program (GIMP) was used to analyze the same samples to assess the differences in the programs. The reproducibility of classification in TWS was tested by training same image 8 times by one user.

The differences in the measurements obtained by BoneJ before and after downsizing the classified images were evaluated to understand the discrepancies in the measurements of trabecular thickness and trabecular separation.

## Stepwise procedures

### Image preparation for segmentation−3 min per image

**Figure 1:**
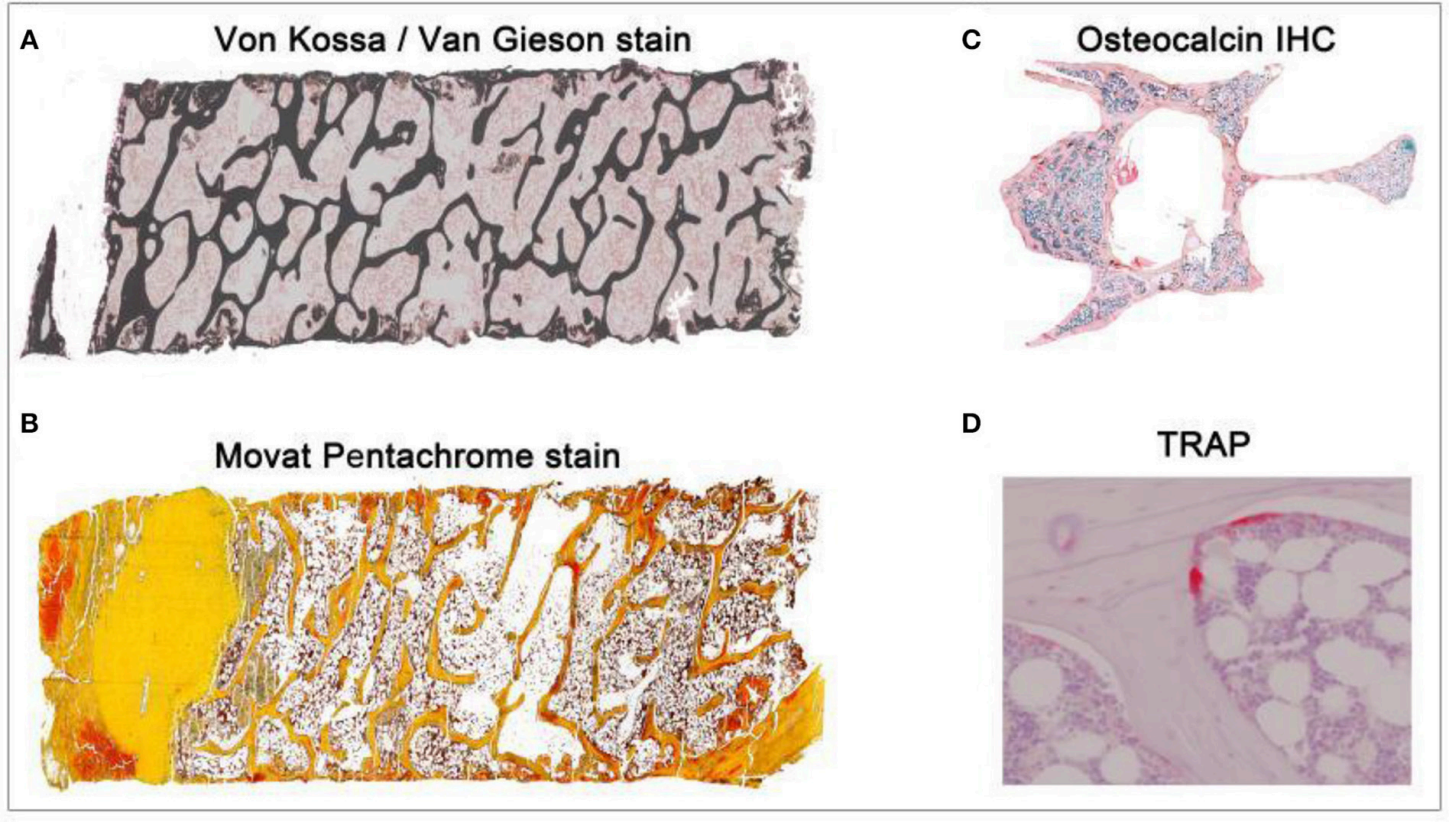
Overview of different histological stains evaluated using TWS and ImageJ toolbox. Sheep iliac crest biopsy and rat lumbar vertebral samples were used to test and set up the protocol. Sheep biopsy samples were embedded in PMMA resin and rat samples were embedded in paraffin. **(A)** Iliac crest sheep biopsy stained with Von Kossa/Van Gieson helped in visualization of mineralized and non-mineralized bone matrix (5X magnification). **(B)** Movat pentachrome stain visualized cartilage, osteoid, and ossified tissue distinctly in sheep sample (5X magnification). **(C)** Osteocalcin IHC visualized the region of osteoblast activity in rat osteoporotic sample (10X magnification). **(D)** TRAP helped in investigating osteoclast activity in the rat bone (40X magnification).

1. Import the image onto ImageJ either using “drag-drop” option or through “Open” option under File drop-down menu.
2. Contour around the bone excluding the muscles part using the “Polygon selection” or “Freehand selection” tool.
3. Clear out the muscles from the image using “Clear outside” under Edit drop-down menu (Figure 1).
4. Divide the whole image into stacks using “Image ➔Stacks ➔Tools ➔Montage to Stack.” The pop-up window will ask the user to input the number of rows and columns to get stacks. In general, 4 X 4 stack size are used to save time during segmentation.
5. Save the stacks as “Image sequence” using “Save as” option from File drop-down menu.

### Trainable Weka segmentation- 15–30 min per image [adapted from (13)]

**Figure 2:**
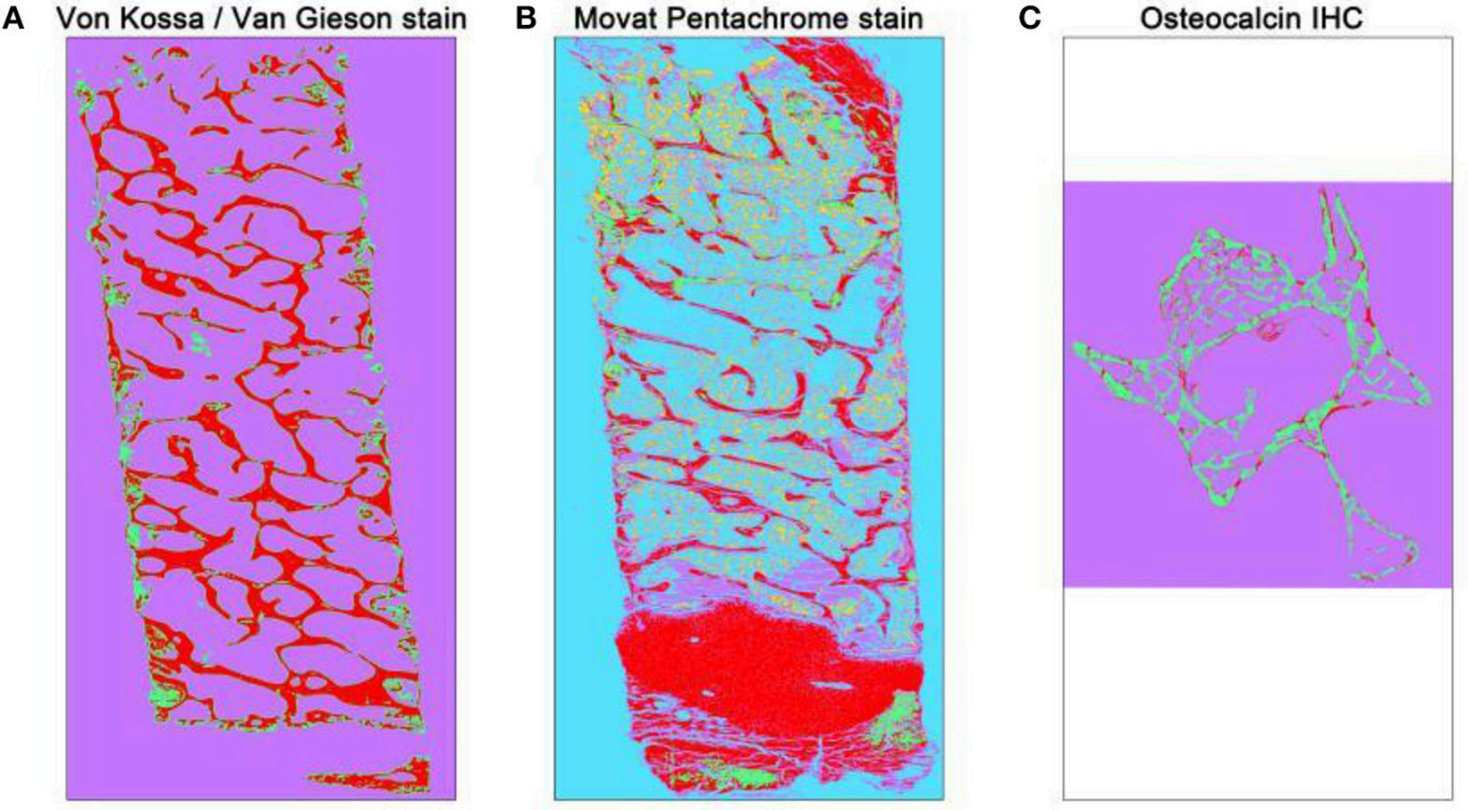
Overview of automated segmented images using TWS. The automated classification was carried out after training one stack of the whole overview image. Different histological stains were trained according to different classes **(A)** Von Kossa/Van Gieson stain classified image depicts mineralized bone matrix as red and non-mineralized bone matrix as green. The magenta color here represents the background class. **(B)** Movat pentachrome stain classified image depicts ossified tissue as red, osteoid (non-mineralized) as green, cartilage as magenta, bone marrow as yellow, and background class as turquoise. **(C)** Osteocalcin classified image depicts osteocalcin positive as red, bone as green, and background as magenta.

1. Select one of the stack images from the previous step that contains all the color/segment of a sample.
  In case of:
  Von Kossa/Van Gieson stain: stack containing mineralized as well as non-mineralized bone matrix.
  Movat Pentachrome stain: stack containing ossified tissue, osteoid, bone marrow, and cartilage.
  Osteocalcin IHC: stack containing osteocalcin positive region and bone region.
2. Import the selected stack using “drag-drop” option or through “Open” option under File drop-down menu.
3. Open the TWS window using “Plugins ➔Segmentation ➔Trainable Weka Segmentation.”
4. Define and rename the classes according to the histological stain being investigated. Go to “Settings” option on TWS window and rename/add classes according to the analysis.
5. Using the freehand tool of ImageJ, define and mark the regions under different classes according to the stain being investigated.
  In case of:
  Von Kossa/ Van Gieson stain: define three classes as “mineralized bone,” “non-mineralized bone,” and “background.” Mark the black stained bone portion under mineralized bone and red portion under non-mineralized bone. Mark the bone marrow and other not-required portion under the background class.
  Movat Pentachrome stain: define five classes as “ossified tissue (yellow),” “osteoid (red),” “cartilage tissue (green),” “bone marrow,” and “background.”
  Osteocalcin IHC: define three classes as “osteocalcin positive,” “bone,” and “background.” Mark the red stained portion under osteocalcin and negative stained bone under bone.
6. Define at least 10–15 points for each class to get accurate results. Using “Add to class” option, the marked area can be defined in classes.
7. Click on “Train classifier” option after defining each class. This might take some time depending upon the size of the image and computer capacity.
8. The log window updates with the each step of segmentation.
9. “Create result” option gets activated as soon as the classification is over. Additionally, the log window also updates when the image segmentation is done.
10. Click on “Create result” and then compare the input image with the result image to confirm the image segmentation results (Figure 2).
11. Save the classifier file after successful segmentation by clicking on “Save classifier” option from TWS window.
12. The saved classifier file can be used later to train the batch of similar stained images.

### Manual histomorphometry of segmented images- 30–50 min per image

1. Import each image manually to the TWS window and upload the saved classifier from previous step.
2. Click on “Train classifier” afterwards.
3. Save result image using “Save result” option.
4. Repeat steps 1–3 until all images are segmented. Close the TWS window afterwards.
5. Upload first result image to the ImageJ. Obtain the area percentage of each pre-defined classes using “Analyze ➔Measure.”
6. Obtain the dimensions of each image by setting up scale. Click on “Set scale” under Analyze drop-down menu. Now, the scale of the image is visible on the top left of the image.
7. Multiply the obtained area percentage with the image scale to obtain the area values in μm or mm.
8. Repeat steps 5–6 until the results are obtained.

However, the long manual process of histomorphometry (as shown above) can be replaced by the automated TWS script discussed in this manuscript. The procedure to carry out the automated histomorphometry is as shown below:

### Automated (modified) histomorphometry of segmented images- 30–40 min per image

1. Create an “Input” folder and copy all the same stained images in it.
2. Copy the “TWS_automated.bsh” script in sub-folder “Utilities” under “Fiji folder ➔Plugins ➔Scripts ➔Plugins ➔Utilities.” Alternatively, the script can be stored in the user-choice sub-folder too. Alternatively, the script can be run using “ImageJ ➔Plugins ➔Macros ➔Run.”
3. Run the script by going to “Plugins ➔Utilities ➔TWS_automated.”
4. The prompt window asks user to direct the script toward “Input directory.” The user must directs the program toward the directory where Input folder is created. Next, the user can direct the program toward “Working directory” where results should be saved. The classifier file saved from TWS step can be uploaded under “Classifier file” window. The image scale can be entered here to obtain the result values in μm or mm accordingly.
5. Click on “OK” after defining the path and scale values. The next prompt window asks for the user input to define stack size. Additionally, the prompt window asks user for “enhance contrast” and “probability maps.”
6. Click on “OK.”
7. The area percentage and area in defined scale values will be saved automatically at the end after all images are analyzed.

### Manual measurement of Tb. Th and Tb.Sp using Bonej: 30–60 min per image

**Figure 3:**
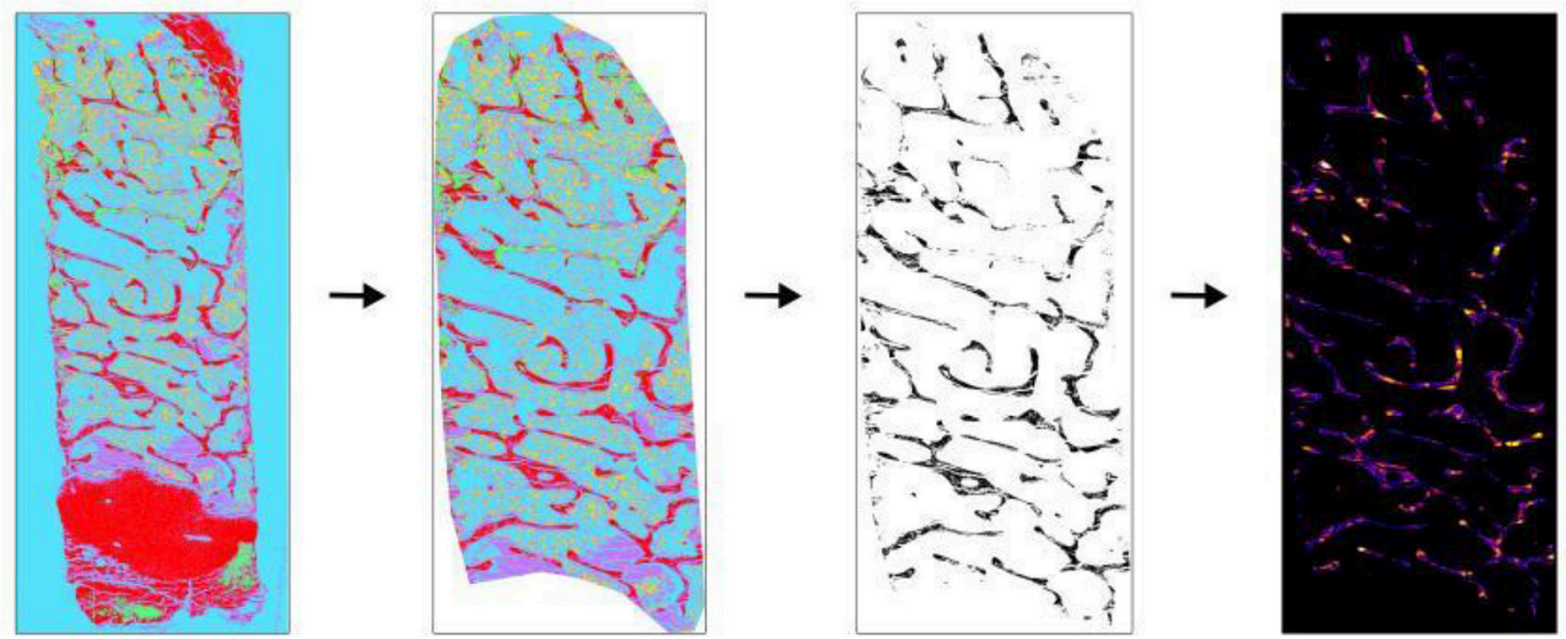
Application of BoneJ in the measurement of Tb.Th and Tb.Sp. Movat pentachrome stain classified overview of sheep iliac crest biopsy was used to obtain Tb.Th and Tb.Sp. (Left to right). The classified overview was uploaded and scale was set. The cortical bone and cartilage area was cleaned out to measure trabecular parameters. The image was then converted into binary using ImageJ toolbox. Trabecular bone appears as black and other as white. “Thickness” option present in BoneJ drop-down menu was selected and output graphical overview along with measured values were obtained.

1. The automated TWS script saves the classified overview images under “Classified overviews” sub-folder in the working directory. The working directory was defined by the user in the previous session.
2. Import the classified image onto ImageJ and remove the cortical bone using “Freehand selection” or “Polygon selection” (Figure 3).
3. Clear out the cortical bone portion from the image using “Clear outside” under Edit drop-down menu.
4. Set scale of the image using “Set scale” under Analyze drop-down menu.
5. Convert the image into 8-bit using “Image ➔Type ➔8-bit”option.
6. Create binary image of the obtained 8-bit image using “Make binary” option from “Process ➔Binary” option. The trabecular bone appears in black and other as white portion.
7. Go to “Plugins ➔BoneJ ➔Thickness” to measure Tb.Th and Tb.Sp [adapted from (14)].
8. A pop-up window asks user to select for thickness and spacing. Check the “spacing” option to get the separation values.
9. The result window will provide the values of Tb.Th and Tb.Sp in the user-defined scale. Additionally, graphical results can be saved.
10. Repeat steps 2–9 until all images are analyzed.

However, the time consuming manual protocol for Tb.Th and Tb.Sp measurements (as shown above) can be replaced by the automated BoneJ script discussed in this manuscript. The procedure to carry out the automated Tb.Th and Tb.Sp measurements are as shown below:

### Semi-automated (modified) measurement of Tb. Th and Tb.Sp using bonej: 2–5 min per image

1. Install “Thickness_seperation_automated.txt” macro by using “Plugins ➔Macros ➔Install.” Alternatively, the user can directly run the script using “Plugins ➔Macros ➔Run.”
2. Run the macro once it is installed.
3. The prompt window asks user to direct the script toward “Input directory.” The input directory in this case is the classified overview folder. The image scale can be entered here to obtain the result values in μm or mm accordingly. The script will by default downsize the image to 0.25 to quickly calculate the values.
4. Click on “OK” and another continuous pop-up windows will come up. Here, the user can define the region of interest (ROI; trabecular bone) to measure Tb.Th and Tb.Sp. The user has to define the ROI for each classified image once.
5. The script will run in the background.
6. The result excel file will be created at the end with the name of the sample and their respective Tb.Th and Tb.Sp values.

### Measurement of osteoclast activity using ImageJ toolbox: 1 min per image

**Figure 4:**
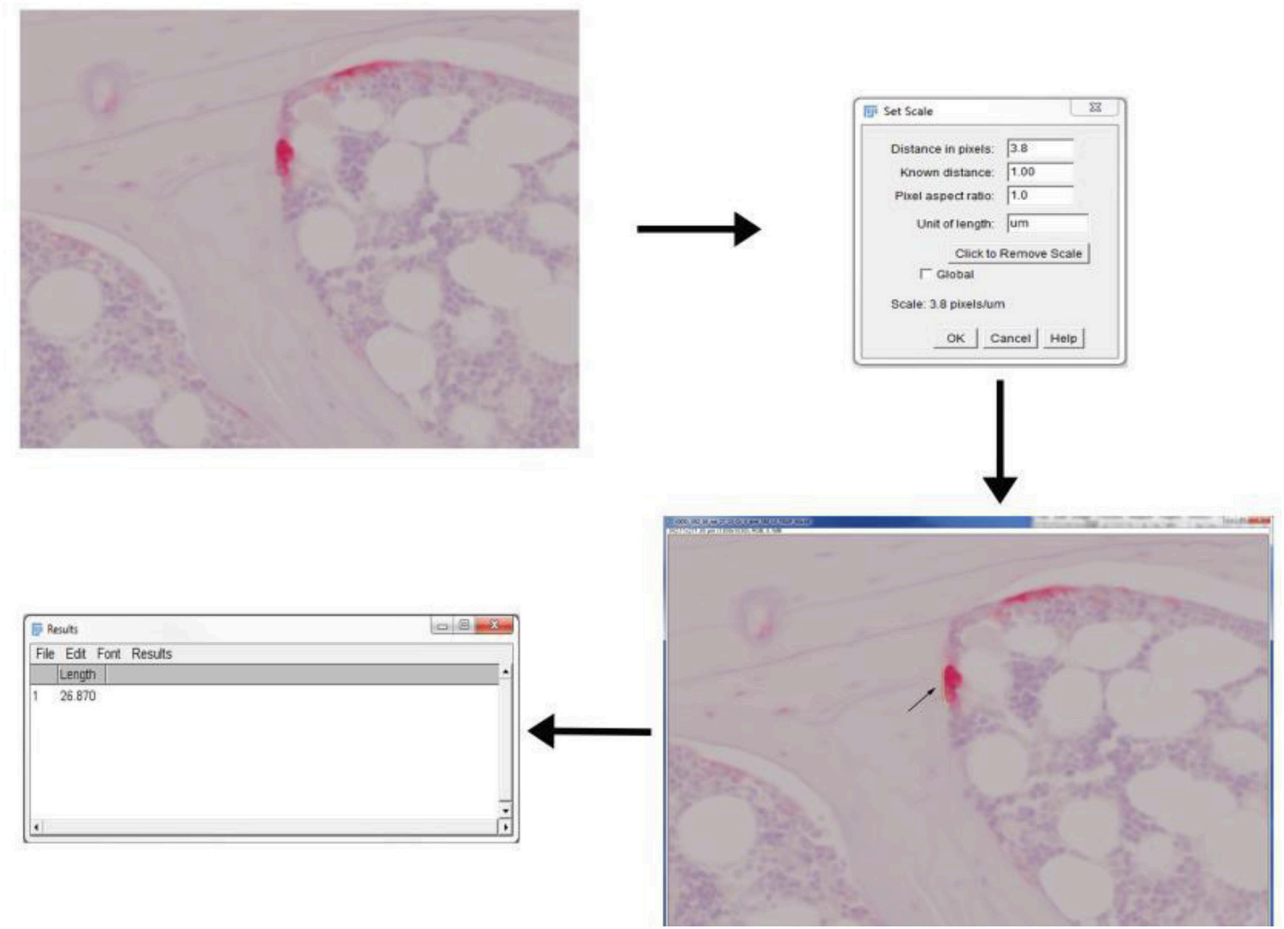
Application of ImageJ toolbox to measure osteoclast activity from TRAP stained sections. Rat vertebral sample was used to perform TRAP enzyme histochemistry. Osteoclasts are identified as multi-nucleated TRAP positive cells near the bone surface. The scale was set up before proceeding with measurements. The length of ruffled borders (arrows) govern the osteoclast activity. The length of ruffled border was measured using free-hand line tool after the scale was set.

1. Import the TRAP stained 40X image onto ImageJ either using “drag-drop” option or through “Open” option under File drop-down menu (Figure 4).
2. Set scale of the image using “Set scale” under Analyze drop-down menu.
3. Osteoclast activity is mainly defined by the count of osteoclast and the length of ruffled border. Therefore, draw a line across ruffled border using “Freehand line” option.
4. Obtain the length of ruffled border by selecting “Measure” option from Analyze drop-down menu.
5. Repeat step 3 and 4 until all images are done.

## Anticipated results

TWS is a machine learning based tool that uses manual annotation to train a classifier and automatically segment the remaining data. TWS can make use of predefined image features. The color based image segmentation plays a critical role in the quantitative evaluation of bone parameters like mineralized and non-mineralized bone matrix area (Tables 1, 2). This protocol using TWS resulted in the area percentage distribution of mineralized and non-mineralized bone matrix in an osteoporotic sheep sample. These area measurements will help in understanding the bone loss. Nonetheless, analysis of immunostainings using TWS helped in monitoring the osteoblast activity across the study (data not shown). Besides the measurement of area, the investigation of trabecular thinning using BoneJ provided a comprehensive overview in our study. Such parameters helps in correlating 2D analysis with 3D analysis in bone research.

**Table 1:** Overview of classes defined in different histological stains.

**Histological stain**	**Classes defined in TWS window**
Von Kossa/Van Gieson stain	Class 1: Mineralized bone Class 2: Non-mineralized bone Class 3: Background
Movat Pentachrome stain	Class 1: Ossified tissue Class 2: Osteoid (non-mineralized matrix) Class 3: Cartilage tissue Class 4: Bone marrow Class 5: Background
Osteocalcin IHC	Class 1: Osteocalcin positive Class 2: Bone Class 3: Background

**Table 2:** Values obtained from the TWS, BoneJ, and ImageJ toolbox protocols (in μm).

	**Histological stain:**	**Class 1**	**Class 2**	**Class 3**	**Class 4**	**Class 5**
TWS	Von Kossa/Van Gieson stain	12.3	6.785	80.915	
	Movat Pentachrome stain	53.832	6.280	23.165	2.685	14.038
	Osteocalcin IHC	0.993	5.252	93.754	
BoneJ	Movat Pentachrome	Trabecular thickness = 23.705 μm Trabecular separation = 888.785 μm				
ImageJ	TRAP	Ruffled border length = 26.870 μm				

### Potential pitfalls and troubleshooting measures

Both the optimized scripts were designed to facilitate quantitative evaluation of histological stained samples and prevent the manual work and time taken for analysis. Although the scripts rely on the latest version of java and Fiji based ImageJ, an unavoidable limitation is the dependence of evaluation time on the computer hardware system. The evaluation time might increase based on the size of sample images and computer capacity. Nonetheless, the automated scripts save high amount of manual work. The results obtained from TWS, BoneJ, and osteoclast activity measurements are shown in Table 2.

The protocol described here, however, has a few limitations as listed below:

1. The slight degree of differences between manual and semi-automated measurements can occur due to user based pixel identification procedure (as in TWS).
2. Parameters like Tb.Th and Tb.Sp relies on the user selected ROI for the measurements by BoneJ. Therefore, slight chances of deviations are present in these measurements by different users. The problem can be resolved by saving the ROI and re-applying it for trabecular measurements from same sample.
3. The classifier file obtained from TWS can be applied only to the same magnification images. However, the classifier file obtained from large magnification pictures can be applied to the smaller magnification.

User might come across few errors or failure messages while working with the ImageJ. Following are the expected errors and possible solution to them:

1. Image too big to import and simultaneous hanging of ImageJ. *Trou*bleshooting: Increase the computer memory assigned to ImageJ by selecting “Edit ➔Options ➔Memory & Threads.” The user should not assign more than half of random access memory to the ImageJ. Restart the ImageJ after memory assignment.
2. The computer system takes a long time or fails to analyze the whole overview images of sample. *Trou*bleshooting*:* The option of “Montage to stack” is added in the protocol to prevent the occurrence of such error. The number of stacks should be made in direct proportion of computer memory.
3. TWS fails to differentiate between two closely related colors and gives false results. *Trou*bleshooting*:* The “enhance contrast” feature should be used prior to image segmentation to overcome such problems.
4. In certain cases, ImageJ results in java based errors in between the trainable weka segmentation. *Trou*bleshooting*:* The established script works with the latest version of java and ImageJ. Therefore, update it regularly.
5. BoneJ error: could not find zip file for the installation of 3D libraries. *Trou*bleshooting*:* User can install 3D libraries manually and copy to plugins folder of ImageJ. Restart the ImageJ and run BoneJ.
6. The conversion of classified image to binary results in bone as white and other as black. This will give the false results.

*Trou*bleshooting*:* Click on “Invert” option under Edit drop-down menu to inverse the colors.

## Limitations

The script used in this protocol is used routinely to successfully quantify the bone parameters. However, there are a few limitations to the application of program as listed below:

There is no possibility to analyze the whole image at one time using TWS without creating the stacks.

In case of histological stain like Toluidine Blue, the bone and the bone marrow are visualized in the same color which makes it difficult for the program to differentiate. Therefore, the bone marrow must be cleared out prior to the TWS.

The optimized BoneJ script fail to provide Tb.Th and Tb.Sp in case of non-homogenous bone sections (for example major cracks). This might result in an outlier.

The optimized scripts fail to automatically count the cells (like osteocytes, osteoblast).

## Discussion

Bone histomorphometry following ASBMR standards provide quantitative information on metabolic bone diseases and fracture healing (1, 22). Histomorphometry is grouped into: static and dynamic histomorphometry. Static histomorphometry involves evaluation of bone parameters at a particular time point while dynamic histomorphometry involves evaluation of bone structure during time series experiment (23). Further, static histomorphometry includes evaluation of parameters like osteoblast, osteoclast activity. While, dynamic histomorphometry includes evaluation of bone mineralization from fluorochrome labeled samples. The standards for both static and dynamic histomorphometry are well-defined. Although micro-computed tomography (micro-CT) and DXA are the gold standards in bone research, histomorphometry is essential to get cellular insight. This will indeed help in bridging a gap between 2D and 3D analysis of bone samples. Intriguingly, Müller R et al. reported significantly higher correlation between histomorphometric and micro-tomographic analysis of human bone biopsies (6). Nonetheless, histology and histomorphometry provides additional information related to the biomarkers activity (IHC) and bone mineralization. Therefore, histomorphometry is one of the building block in bone research.

The global application of common histomorphometry methods to analyze different set of images are much needed. Previous studies reported different concerns about application of semi-automated or automated software for bone histomorphometry (24). The need of standardized algorithm which prevents any interference with quantification procedure during analysis is needed. However, the use of complicated algorithm and protocols makes it difficult for routine use in preclinical and clinical research. Hence, there is an urgent need of setting up an easier and user-friendly bone histomorphometry method.

Our study focused on establishing an automated easily accessible scripts linked to ImageJ to perform bone histomorphometry. TWS tool developed by Arganda-Carreras et al. (13) was adapted and further improved to perform automated bone histomorphometry. The method described in our study provides user-friendly boundary without any need of programming experience. The manual segmentation method for analyzing each single image was time-consuming. The used program was applied before to analyze mineralized and non-mineralized bone matrix from Trichrome Masson Goldner stain (15). The script was applied to several different stains like toluidine blue, Von Kossa/Van Gieson and immunostainings (smooth muscle actin, osteocalcin, alkaline phosphatase). Besides, the script was successfully tested on different magnification pictures. This automated segmentation of images saved the analysis time. The trained classifier from higher magnified images can be applied to lower magnification pictures but not vice-versa. Also, this script provides you with first whole classified image to assure complete transparency in the working pipeline.

Polig et al. applied computer controlled microphotometric method to obtain measurements of bone parameters like percentage of bone, trabecular thickness (8). They manually scanned the fluorochrome labeled dog specimen followed by the measurement of light intensity using photomultiplier. However, such complex procedure lacks global application in bone research. Our established workflow on the contrary, works on tile scan images as well as on specific ROI from whole histological sample. This indeed saves much of user time and prevents chances of false positives.

Zhang et al. implemented Visiopharm algorithm to analyze bone, cartilage, and fibrous tissue area from histological section of murine femoral allografts (9). Visiopharm assigns the class label for all tissue in a stain and user can choose for the batch processing for the consecutive samples from same stain. However, Visiopharm application was only limited to the fracture healing studies in murine model. On the contrary, our established scripts were used broadly on sheep, rat, murine, and human samples from different studies. Besides, Visiopharm is a commercially available software for bone histomorphometry while our scripts are freely available. Thus, the open source feature of our plug-in makes it more suitable for increased application.

van't Hof et al. established an open-source ImageJ based programs to measure features like osteoclast area, mineralized bone from mouse lumbar spine and human iliac crest biopsies (11). They established three different programs; TrapHisto, OsteoidHisto, and CalceinHisto to perform respective histomorphometry following ASBMR guidelines. In addition, the program provided an option to remove the sectioning artifacts like cracks. While, our implemented script provides no option to remove such artifacts. Indeed, the user must remove such artifacts prior to analysis. However, the program developed by van't Hof et al. (11) requires continual attention while measuring the bone parameters to prevent errors. Our TWS script requires user attention only during the initial set up time during image preparation for segmentation step.

**Figure 5:**
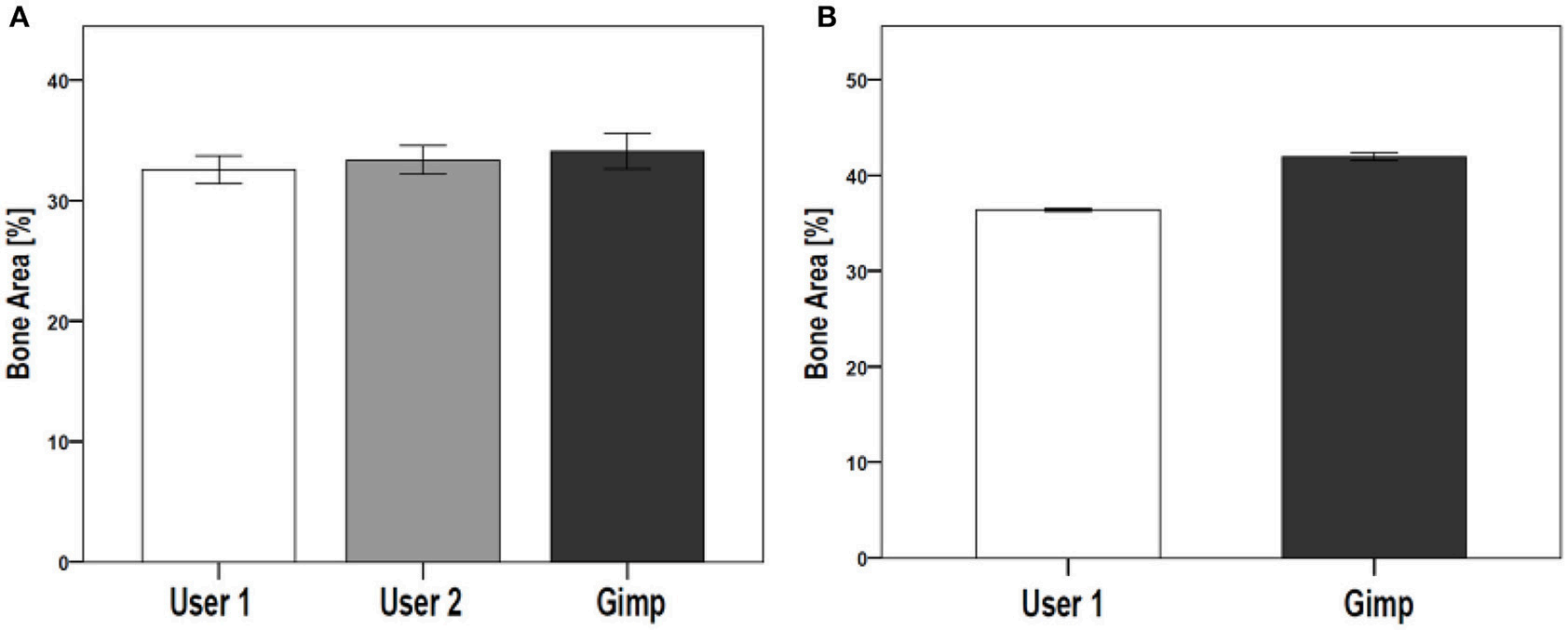
Comparison of TWS results analyzed by two different users and GIMP image analysis software. The reproducibility and validation of generated TWS script was tested by investigating inter-observer differences. **(A)** Two users were given same set of images to analyze using TWS. Alongside, GIMP based analysis was carried out by one of the user. The analysis was carried out on rat lumbar vertebrae hematoxylin stained sections (data not shown here). No significant differences were seen. **(B)** The variation in image classification was further tested by giving one image for analysis to a user. The user performed classification using TWS for eight different times. The results showed no large fluctuations in the obtained values. The similar analysis was carried out using Gimp, which showed higher values compared with TWS results. [*N* = 9 for **(A)**, the graphs were plotted as Mean ± Standard error of mean].

The reproducibility of the semi-automated TWS was examined in a blinded experiment with two users on two different workstations (Figure 5A). Furthermore, the comparability and accuracy was then examined by testing the semi-automated TWS against the manual histomorophometrical analysis in GIMP by user one on the same workstation. The variations of the bone area/total area percentage were not significantly different neither between the two users nor the two programs (Table 3). These variations direct toward differences in user interpretation and robustness of the semi-automated procedure against the manual selection according to experience. Furthermore, the accuracy of classification in TWS was checked by classifying single image eight different times by the user one (Table 4). The obtained bone area percentage values showed no significant differences (Figure 5B), thereby reflecting on the reproducibility and accuracy of TWS over manual GIMP analysis. The established workflow of TWS used in this study was also used previously in osteoporosis study and helped in correlating the results obtained from radiological data and molecular analysis (15).

**Table 3:** Bone area measurements using TWS by two different users and GIMP analysis (in %).

**No. of samples**	**User 1**	**User 2**	**Using GIMP**
1.	29.05019	*31.296849*	38.0032572
2.	*32.443313*	*32.287476*	34.8598266
3.	*32.499343*	*32.839497*	33.9143291
4.	*37.07252*	*35.641512*	39.0762659
5.	*33.560978*	*34.14654*	39.0127477
6.	*36.078188*	*39.451144*	33.4931045
7.	*31.927108*	*32.930072*	25.4887238
8.	*34.508112*	*35.279444*	30.5129929
9.	*25.901145*	*26.463556*	32.5495623
Mean	*32.5601*	*33.370677*	34.1012011

**Table 4:** Bone area measurements obtained after classifying same image but at different times on TWS and GIMP (in %).

**No. of repetitions**	**User 1**	**Using GIMP**
1.	36.096926	42.441
2.	*36.923992*	41.325
3.	*37.112944*	41.899
4.	*36.457707*	41.935
5.	*36.054855*	44.344
6.	*36.324158*	41.648
7.	*35.831968*	41.136
8.	*36.325138*	40.764
Mean	*36.390961*	41.9365

The manual and semi-automated protocol of BoneJ were tested to assess variations in the Tb.Th and Tb.Sp values. The generated script utilizes the feature of “downsizing” to quickly analyze the images. The downsizing parameter (0.25 in this script), however, was set up after trial and testing on set of images. This assured the prevention of false positive values. In this protocol, we showed the resulted values of Tb.Th using manual protocol and semi-automated script (Table 5). Additionally, the Tb. Th measurements were carried out for the biological replicates used in this protocol (Table 6).

**Table 5:** Tb.Th measurement with and without downsizing option (in μm).

**Image**	**Tb.Th**	**Tb.Th (SD)**	**Tb.Th (max)**
Using manual protocol	23.705	19.887	105.091
Using semi-automated protocol	23.940	19.882	105.091

**Table 6:** Tb.Th measurement for the biological replicates of the test sample (in μm).

**Samples**	**Tb.Th**	**Tb.Th (SD)**	**Tb.Th (max)**
Replicate 1	122.154	66.948	349.445
Replicate 2	138.961	78.770	396.141
Replicate 3	159.769	82.557	374.550
Replicate 4	111.001	53.460	272.000
Replicate 5	121.837	69.284	345.393
Replicate 6	151.032	83.250	397.432

Taken together, we believe our scripts will be useful to the scientific community. The scripts rely upon an updated Java and ImageJ version and thusly run on Apple Macintosh and Linux systems without any change. The software described here will run on PCs with at least 4GB of RAM and 64-bit operating system. The source code of script is freely available in Supplementary Data. Users with sufficient programming skills can thus extend the code according to their requirements. The open access to the source code, thus keep the data transparency in research.

## Conclusion

Automated bone histomorphometry script is available for everyone to download, use, and modify freely. The scripts calculate several parameters in user-friendly and convenient format. The measurements are made according to the standardized nomenclature, thusly allowing the increase use in scientific community.

## Author contributions

All authors listed have made a substantial, direct and intellectual contribution to the work, and approved it for publication.

### Conflict of interest statement

The authors declare that the research was conducted in the absence of any commercial or financial relationships that could be construed as a potential conflict of interest.
